# Molecular Typing of Environmental and Clinical Strains of *Vibrio vulnificus* Isolated in the Northeastern USA

**DOI:** 10.1371/journal.pone.0083357

**Published:** 2013-12-30

**Authors:** Yann Reynaud, Steven Pitchford, Sophie De Decker, Gary H. Wikfors, Christopher L. Brown

**Affiliations:** NOAA (National Oceanic and Atmospheric Administration), NEFSC (Northeast Fisheries Science Center), Milford Laboratory, Milford, Connecticut, United States of America; University of Mississippi Medical Center, United States of America

## Abstract

*Vibrio vulnificus* is a ubiquitous marine bacterium that is responsible for infections and some seafood-related illnesses and deaths in the United States, mainly in individuals with compromised health status in the Gulf of Mexico region. Most phylogenetic studies focus on *V. vulnificus* strains isolated in the southern United States, but almost no genetic data are available on northeastern bacterial isolates of clinical or environmental origin. Our goal in this study was to examine the genetic diversity of environmental strains isolated from commercially-produced oysters and in clinical strains of known pathogenicity in northeastern United States. We conducted analyses of a total of eighty-three strains of *V. vulnificus*, including 18 clinical strains known to be pathogenic. A polyphasic, molecular-typing approach was carried out, based upon established biotypes, vcg, CPS, 16S rRNA types and three other genes possibly associated with virulence (arylsulfatase A, *mtlABC*, and *nanA*). An established Multi Locus Sequence Typing (MLST) method was also performed. Phylogenetic analyses of these markers and MLST results produced similar patterns of clustering of strains into two main lineages (we categorized as ‘LI’ and ‘LII’), with clinical and environmental strains clustering together in both lineages. Lineage LII was comprised primarily but not entirely of clinical bacterial isolates. Putative virulence markers were present in both clinical and environmental strains. These results suggest that some northeastern environmental strains of *V. vulnificus* are phylogenetically close to clinical strains and probably are capable of virulence. Further studies are necessary to assess the risk of human illness from consuming raw oysters harvested in the northeastern US.

## Introduction

*Vibrio vulnificus* is a gram-negative bacterium ubiquitous in estuaries and marine coastal environments throughout the world. This species can be isolated from oysters and other shellfish, fish, zooplankton and crabs. *V. vulnificus* can be highly pathogenic to humans, causing wound infection and primary septicemia; infection can lead to 50% mortality in susceptible hosts (individuals with compromised immune function resulting from liver disease, diabetes, cancer, hemochromatosis, immune depression, and other chronic conditions [1]). *V. vulnificus* currently represents the leading cause (95%) of seafood-related death in the United States [2]–[4], mainly as a consequence of consumption of raw oysters. According to the Centers for Disease Control and Prevention (CDC), approximately 100 persons are infected by *V. vulnificus* each year in the USA, mainly in southern states, with the highest incidence in the Gulf of Mexico region during summer.

Biotyping, based upon biochemical, serological, and genetic properties, and host range, allows the categorization of three biotypes in the *V. vulnificus* species. Biotype 1 strains constitute the majority of bacteria responsible for human infections [5]. Biotype 2 strains are primarily eel pathogens, but some cases of human infection have been caused by biotype 2 serovar E [6]–[8]. Biotype 3 is a mosaic hybrid and the only biotype associated with human outbreaks in Israel [9].

Many molecular typing approaches have been applied to clinical and environmental *V. vulnificus* strains in previous studies, allowing the characterization of alternative genotypes. Polymorphism in 16S rRNA sequences has led to the categorization of types A and B [5], [10]–[14], with significant correlations associating clinical strains with type B and environmental strains with type A. Similar genetic differentiation with two main lineages is obtained with capsule CPS alleles 1 and 2 of the capsule group 1 CPS operon [5], [15], with vcg (a virulence-correlated gene first identified by Random Amplified Polymorphic DNA (RAPD-PCR)) types C and E [5], [14], [16], [17]. Multi Locus Sequence Typing (MLST) was also developed using both housekeeping and virulence genes sequences; as with the other typing approaches, two main clusters were found, but sub-clusters were also highlighted and correlated to varying degrees with clinical status and biotypes [6], [14], [18].

Despite statistical correlations between genotypes and strains of clinical or environmental origin, genotype is not diagnostic, i.e., genotype cannot predict unequivocally the virulence of an isolate [5]. No available molecular marker has sufficient resolving power to categorize with absolute certainty the pathogenicity of a strain. Nevertheless, complete genome sequencing of *V. vulnificus* strains belonging to both genotypes, and from clinical or environmental sources [19], [20], allowed the identification of genes potentially useful as markers of virulence that can be tested in parallel with virulence tests using the mouse bioassay model.

In the northeastern United States, far fewer cases of human infection are reported each year than in the South [4]. Samples of *V. vulnificus* can be isolated from the environment and from oysters during summer [21]–[23], but we are unaware of any molecular typing study of clinical and environmental *V. vulnificus* strains isolated from oysters from the northeastern USA. Our goal in the present study was to examine the genetic diversity of *V. vulnificus* strains from oysters in the northeastern USA and to compare such environmental isolates with clinical strains. A polyphasic molecular-typing approach was developed based upon: 1) 16S rRNA, CPS, vcg, biotyping, 2) MLST on housekeeping and virulence genes, and 3) the presence or absence of putative virulence markers by PCR. This is the first study aiming at a genetic comparison of clinical and environmentally sourced strains in northeastern USA.

## Materials and Methods

### Sample collection

Eighty-three *V. vulnificus* strains were examined (Table 1). Of these strains, 18 were recovered from clinical sources (blood, wound infection, stool, and gall bladder) provided by the CDC and 60 were isolated from specimens of the Eastern Oyster *Crassostrea virginica*. The complete sequences of 5 other strains (CMCP6, YJ016, MO-24/O, JY1701 and JY1305) already published [19], [24]–[26] were used for the MLST analysis. Clinical strains were isolated between 2003 and 2010 from Atlantic coastal areas in the eastern USA (between Massachusetts and South Carolina), and environmental strains from oysters were isolated during the summer of 2012 when the temperature exceeded 21°C in coastal Maine, Massachusetts, New Hampshire, Rhode Island, New Jersey, Virginia and South Carolina. No specific permissions were required for oyster sampling and field studies did not involve endangered or protected species. Indeed, oysters are not covered by the Animal Welfare Act, so they are exempt from oversight by any Animal Care and Use Committee (IACUC). The oysters were sent voluntarily by oyster growers who produce them on private or leased plots. Oyster homogenates were prepared as described previously [27], and putatively-identified *V. vulnificus* strains were selected on the specific medium CPC+ [28]. All strains were confirmed as *V. vulnificus* by PCR targeting the hemolysine gene *vvhA* after boiling 10 min at 100°C as previously described [29]. All strains were grown on Marine Broth 2216E (Difco) at 37°C and stored frozen at −80°C in 30% (v/v) glycerol/Marine Broth.

**Table 1:** Characteristics and molecular typing of the *V. vulnificus* strains used in this study.

Strain name	Source^a^	Tissue	State or country of isolation	Month/Year of isolation	vcg type	CPS type	16S rRNA type	PCR multiplex biotype	MLSTlineage	PCR Aryl-sulfatase	PCR *mtlABC*	PCR *nanA*
2448-03	C	Blood	VA	07/2003	E	2	A	1	I			+
2010V-1021	C	Stool	MD	04/2010	E	2	A	1	I		+	+
AM 37219	C	Blood	VA	08/2008	E	2	A	1	I		+	
AM 40459	C	Blood	MD	06/2009	C	0	B	1	II	+	+	+
AM 41118	C	Gall Bladder	MA	08/2009	C	1	B	1	II	+	+	+
AM 41299	C	Blood	NC	11/2009	C	1	B	1	II	+	+	+
AM 41394	C	Blood	CT	09/2009	E	2	A	1	I		+	+
AM 41396	C	Blood	CT	09/2009	E	2	A	1	I	+	+	+
AM 41493	C		MD	10/2009	C	2	A	1	I			+
AM 41942	C	Blood	VA	11/2009	C	1	B	1	II	+	+	+
AM 42265	C	Wound	SC	08/2009	C	2	B	1	II	+	+	+
AM 43809	C	Blood	VA	06/2010	C	2	A	1	II	+	+	+
AM 45386	C	Wound	MD		E	2	A	1	I		+	+
AM 45637	C	Blood	NC	07/2010	E	2	A	1	I	+	+	+
AM 45926	C		CT	10/2010	E	2	A	1	I	+	+	+
AM 46215	C	Blood	VA	10/2010	E	2	A	1	I	+	+	+
AM 46407	C	Blood	SC	Mai 2010	E	2	A	1	I	+	+	+
AM 46459	C	Stool	MD	06/2010	C	2	A	1	I	+	+	+
VvMBC26	E	Oyster	NJ	06/2012	E	2	A	1	I			
VvMBC27	E	Oyster	NJ	06/2012	E	2	A	1	I			
VvMBC28	E	Oyster	NJ	06/2012	E	2	A	1	I			+
VvMBC29	E	Oyster	NJ	06/2012	E	2	A	1	I			+
VvMBC30	E	Oyster	NJ	06/2012	E	2	A	1	I			+
VvMBC31	E	Oyster	NJ	06/2012	E	2	A	1	I	+		+
VvMBC32	E	Oyster	MA	06/2012	E	0	A	1	I		+	
VvMBC33	E	Oyster	NJ	06/2012	E	2	A	1	I			+
VvMBC34	E	Oyster	NJ	06/2012	E	0	A	1	I			
VvMBC35	E	Oyster	NJ	06/2012	E	2	A	1	I			
VvMBC36	E	Oyster	VA	07/2012	E	2	A	1	I			+
VvMBC38	E	Oyster	VA	07/2012	E	0	A	1	I		+	
VvMBC41	E	Oyster	VA	07/2012	E	0	A	1	I		+	
VvMBC44	E	Oyster	VA	07/2012	E	2	A	1	I			
VvMBC50	E	Oyster	VA	07/2012	E	2	A	1	I			
VvMBC51	E	Oyster	VA	07/2012	E	2	A	1	I			
VvMBC52	E	Oyster	NJ	07/2012	E	0	A	1	I		+	
VvMBC53	E	Oyster	NJ	07/2012	E	0	A	1	I		+	
VvMBC54	E	Oyster	NJ	07/2012	E	0	A	1	I		+	+
VvMBC55	E	Oyster	NJ	07/2012	E	1	A	1	I		+	
VvMBC56	E	Oyster	NJ	07/2012	E	1	A	1	I	+	+	+
VvMBC57	E	Oyster	NJ	07/2012	E	1	A	1	I	+	+	+
VvMBC58	E	Oyster	NJ	07/2012	E	2	A	1	I		+	
VvMBC59	E	Oyster	NJ	07/2012	E	2	A	1	I		+	
VvMBC60	E	Oyster	NJ	07/2012	E	2	A	1	I		+	
VvMBC61	E	Oyster	NJ	07/2012	E	1	A	1	I		+	
VvMBC62	E	Oyster	NJ	07/2012	E	1	A	1	I		+	
VvMBC63	E	Oyster	NJ	07/2012	E	1	A	1	I			
VvMBC64	E	Oyster	NJ	07/2012	E	2	A	1	I			
VvMBC65	E	Oyster	NJ	07/2012	E	1	A	1	I			
VvMBC67	E	Oyster	NJ	07/2012	E	2	A	1	I		+	
VvMBC68	E	Oyster	NJ	07/2012	E	2	A	1	I			
VvMBC69	E	Oyster	NJ	07/2012	E	2	A	1	I		+	
VvMBC70	E	Oyster	NJ	07/2012	E	2	A	1	I		+	+
VvMBC71	E	Oyster	NJ	07/2012	E	1	A	1	I		+	
VvMBC72	E	Oyster	NJ	07/2012	E	2	A	1	I	+	+	
VvMBC73	E	Oyster	NJ	07/2012	C	2	A	1	II	+	+	+
VvMBC74	E	Oyster	NJ	07/2012	E	1	A	1	I		+	+
VvMBC75	E	Oyster	VA	07/2012	E	2	A	1	I		+	
VvMBC76	E	Oyster	VA	07/2012	E	2	A	1	I			
VvMBC77	E	Oyster	VA	07/2012	E	0	A	1	I			
VvMBC80	E	Oyster	VA	07/2012	E	2	A	1	I			
VvMBC81	E	Oyster	VA	07/2012	E	2	A	1	I		+	+
VvMBC83	E	Oyster	VA	07/2012	C	0	A	1	I	+	+	+
VvMBC84	E	Oyster	VA	08/2012	E	2	A	1	I		+	
VvMBC85	E	Oyster	VA	08/2012	E	2	A	1	I		+	+
VvMBC86	E	Oyster	VA	08/2012	E	0	A	1	I		+	
VvMBC87	E	Oyster	VA	08/2012	E	0	A	1	I			
VvMBC88	E	Oyster	VA	08/2012	E	2	A	1	I		+	+
VvMBC89	E	Oyster	VA	08/2012	E	1	A	1	I		+	+
VvMBC93	E	Oyster	NH	09/2012	E	2	A	1	I	+	+	+
VvMBC94	E	Oyster	NH	09/2012	E	0	A	1	I	+	+	+
VvMBC97	E	Oyster	NH	09/2012	E	2	A	1	I	+	+	+
VvMBC99	E	Oyster	NJ	09/2012	E	2	A	1	I	+	+	+
VvMBC100	E	Oyster	NJ	09/2012	E	0	A	1	I		+	+
VvMBC101	E	Oyster	VA	09/2012	E	2	A	1	I			+
VvMBC102	E	Oyster	VA	09/2012	E	2	A	1	I		+	
VvMBC103	E	Oyster	VA	09/2012	E	2	A	1	I		+	
VvMBC104	E	Oyster	VA	09/2012	E	2	A	1	I		+	
VvMBC105	E	Oyster	VA	09/2012	E	2	A	1	I		+	
CMCP6	C	Blood	South Korea	2003	C	1	B	1	II	+	+	+
YJ016	C	Blood	Taiwan	1993	C	2	B	1	II	+	+	+
MO6-24/O	C	Blood	US	1986	C	1	B	1	II	+	+	+
JY1701	E	Oyster	US	1999	E		A	1	I			
JY1305	E	Oyster	US	1999	E		A	1	I			

^a^ C, clinical; E, environmental; strains isolated from the same sampling are indicated between lines; + indicate positive PCR signal of arylsulfatase A, MtlABC and NanA coding genes.

### Genotyping

Bacterial DNA samples were extracted and purified as described elsewhere [30]. Molecular typing was performed using three previously-described PCR typing routines targeting genes for: 1) virulence-correlated gene vcg type C or E [16], 2) capsule CPS type 1, 2 or 0 [15], and 3) biotype 2 [7]. Additional PCRs were developed targeting genes potentially useful as markers of virulence [14], [20]. Specifically, sequences coding arylsulfatase A, MtlABC (PTS system mannitol-specific transporter subunit IIC), and NanA (N-acetylneuraminate lyase) using the following, respective, primers: VvNanAF (GCGGTGATCGATCAAATTGCTG), VvNanAR (CCCTTGGTTGAACGCCTCAAT), VvMtlABCF (GCCCAACATCGGGGCATTTA), VvMtlABCR (GGCCAGCTTCTGAAGCCTG), VvArylAF (CCAGACCCGAGCGGATATGC), and VvArylAR (GCGTGTGCGGGCCCCAGA). Further, an MLST approach was applied based upon 6 chromosomal genes used in previous studies [6], [11], [14], [18]: four housekeeping genes (16Sr RNA, *rpoD*, *gyrB* and *glp*), and two genes involved in the virulence of *V. vulnificus* (*pilF* and *vvhA*). All genes except *vvhA* are located on chromosome I. Gene fragments for each of the genes were amplified by PCR (Mastercycler Pro, Eppendorf) using the proof-reading polymerase pfu (G-Biosciences) following manufacturer instructions. PCR products were purified using the QIAquick PCR purification kit (Qiagen). Cycle sequencing reactions were performed using the BigDye Terminator v3.1 Cycle Sequencing Kit (Applied Biosystems). Separation of the DNA fragments was carried out in an API PRISM 3130xl Genetic Analyzer (Applied Biosystems). Nucleotide sequence data reported are available in the GenBank databases under the accession numbers: KF158715 to KF158792, KF241024 to KF241101, KF255160 to KF255393, KF268470 to KF268587 and KF277069 to KF277143.

### Phylogenetic analysis

Concatemers of MLST sequences were aligned with BioEdit [31] using CLUSTAL W [32], and phylogenetic trees were built using the program Phylowin, applying the neighbor joining method and Kimura's 2-parameter distances [33]. Reliability of topologies was assessed by the bootstrap method with 1,000 replicates. Rates of synonymous substitutions per synonymous site (ds), nonsynonymous substitutions per nonsynonymous site (dn), and variance were calculated by the method of Nei and Gojobori [34] using the SNAP program [35] for all genes except 16S rRNA. The ratio of synonymous to nonsynonymous substitutions (ds/dn) was also determined. The nucleotidic diversity index π per-site basis was calculated using the program DnaSP v5 [36].

The ds/dn ratio and π were determined for each gene in all strains, within housekeeping genes and virulence genes, within strains from clinical and environmental origins, and within each major lineage highlighted by MLST (Tables 2 and 3).

**Table 2:** Genetic diversity parameters for the genes used in the MLST.

Gene	Sequence length bp	Polymorphic sites nt	Alleles number	ds^a^	dn^a^	ds/dn ratio	π^a^ nucleotidic diversity per-site basis
16S rRNA	387	15	4	-	-	-	6.1×10^−3^±1.8×10^−3^
glp	591	56	41	7.3×10^−2^±1.4×10^−2^	4.7×10^−4^±2.3×10^−4^	33.3	1.5×10^−2^±1.2×10^−3^
gyrB	633	64	41	6.9×10^−2^±1.2×10^−2^	1.4×10^−3^±8.5×10^−4^	38.8	1.5×10^−2^±1.7×10^−3^
rpoD	930	59	39	4.6×10^−2^±8.1×10^−3^	2.4×10^−3^±9.4×10^−4^	14.3	1.1×10^−2^±1.9×10^−3^
pilF	528	63	41	1.2×10^−1^±1.9×10^−2^	5.6×10^−3^±3.1×10^−3^	19.5	2.6×10^−2^±2.8×10^−3^
vvhA	761	75	43	5.7×10^−2^±8.8×10^−3^	3.5×10^−3^±1.2×10^−3^	14.1	1.5×10^−2^±2.4×10^−3^
House keeping	2541	194	73	6.0×10^−2^±6.2×10^−3^	1.6×10^−3^±4.7×10^−4^	38.7	1.2×10^−2^±1.4×10^−3^
Virulence	1289	138	69	7.8×10^−2^±8.7×10^−3^	4.3×10^−3^±1.3×10^−3^	20.4	1.9×10^−2^±2.3×10^−3^
All	3830	332	81	6.6×10^−2^±5.0×10^−3^	2.6×10^−3^±5.6×10^−4^	36.5	1.4×10^−2^±1.6×10^−3^

Dn, nonsynonymous mutations; ds, synonymous mutations;

^a^ mean ± standard deviation; nt, nucleotides; bp, base pair.

**Table 3:** Comparison of genetic parameters of lineages LI and LII from MLST, and of clinical and environmental strains.

Strains	No strains	Polymorphic sites nt	Alleles number	ds^a^	dn^a^	ds/dn ratio	π^a^ nucleotidic diversity per-site basis
C	21	254	21	1.1×10^−1^±8.3×10^−3^	4.7×10^−3^±1.1×10^−3^	28.4	2.4×10^−2^±2.0×10^−3^
E	62	259	60	4.4×10^−2^±4.1×10^−3^	1.5×10^−3^±3.4×10^−4^	41.4	9.3×10^−3^±8.9×10^−4^
LII	10	192	10	7.1×10^−2^±6.1×10^−3^	2.9×10^−3^±6.9×10^−4^	32.5	1.7×10^−2^±2.6×10^−3^
LI	73	213	70	4.0×10^−2^±3.9×10^−3^	1.3×10^−3^±3.0×10^−4^	41.2	8.4×10^−3^±4.9×10^−4^

Dn, nonsynonymous mutations; ds, synonymous mutations;

^a^ mean ± standard deviation; nt, nucleotides; C, clinical; E, environmental.

In order to investigate the contribution of both point mutation and homologous recombination to *V. vulnificus* isolates evolution under the clonal frame hypothesis, we applied a standard neutral coalescent model using Clonal Frame software version 1.1 [37] specifically designed for MLST data. Inference was performed in a Bayesian framework implemented using Markov chain Monte Carlo (MCMC) sampling. We calculated 50% majority-rule consensus trees based on 100000 iterations (including 50000 burn-in iterations) with initial value of mutation rate θ = 5 per site and two tests with recombination rate ρ = 5 and ρ = 10. Different population-wide evolutionary parameters were calculated: the ratio r/m of probabilities that an individual nucleotide will be altered through recombination and point mutation [38], the ratio ρ/θ of rates at which recombination and mutation occur [39]. A Gelman and Rubin convergence test [40] was performed using Clonal Frame for each parameter. A null hypothesis of recombination was also tested with 100000 iterations. Such analyses were performed for concatenated sequences of whole strains, LI and LII.

Sequences of the three putative virulence markers coding arylsulfatase A, MtlABC, and NanA were also aligned, and phylogenetic trees were built using the same methods by neighbor joining, and using Clonal Frame (100000 iterations, θ = 5 and ρ = 5).

## Results and Discussion

We analyzed a collection of 83 *V. vulnificus* strains (Table 1). Among them, 21 were recovered from clinical sources (18 provided by the CDC, and the 3 complete genome sequences of *V. vulnificus* strains CMCP6, YJ016 and MO6-24O available for *in silico* analysis); 62 recovered from environmental settings (60 strains sampled from oysters during summer 2012 and 2 complete genome sequences of *V. vulnificus* strains JY1701 and JY1305). Based upon multiplex PCR results, no strains belonging to biotype 2 were evaluated in this study; only strains belonging to biotypes 1 or 3; most probably all were biotype 1, considering that biotype 3 is a mosaic hybrid isolated only in Israel [9] and that such strains are easily highlighted by recombination tests such as the ones done in this study. Here no evidence of such recombination events were indicated by our analysis (see below).

### Nucleotidic polymorphism and diversity

The sequences of housekeeping and virulence genes ranged between 387 bp for 16S rRNA and 930 bp for *rpoD*. We first analyzed the nucleotidic polymorphism for each gene used in the MLST analysis (Table 2); it ranged between 15 sites for 16S rRNA to 75 sites for *vvhA*. The nucleotidic diversity π obtained for each gene was comparable to and consistent with values obtained in other MLST studies on *V. vulnificu*s [6], [14], [18]. In the present study, *pilF* showed the highest level of nucleotidic diversity (0.026), and 16S rRNA had the lowest (0.0061). As expected, we obtained higher π values for the two virulence genes *vvhA* and *pilF* (0.019) than for the more-conserved housekeeping genes (0.012) [6].

The ratio of synonymous to non-synonymous substitutions (ds/dn) allowed us to determine the type and level of selection acting on genes. A ratio smaller than 1 indicates diversifying selection, and a ratio higher than 1 indicates purifying selection. All genes analyzed in the present study present a purifying selection with ds/dn ratio much higher than 1 (range between 14.1 for *vvhA* and 38.8 for *gyrB*). These results are consistent with other MLST studies of *V. vulnificus* [14], [18].

### MLST analysis

**Figure 1:**
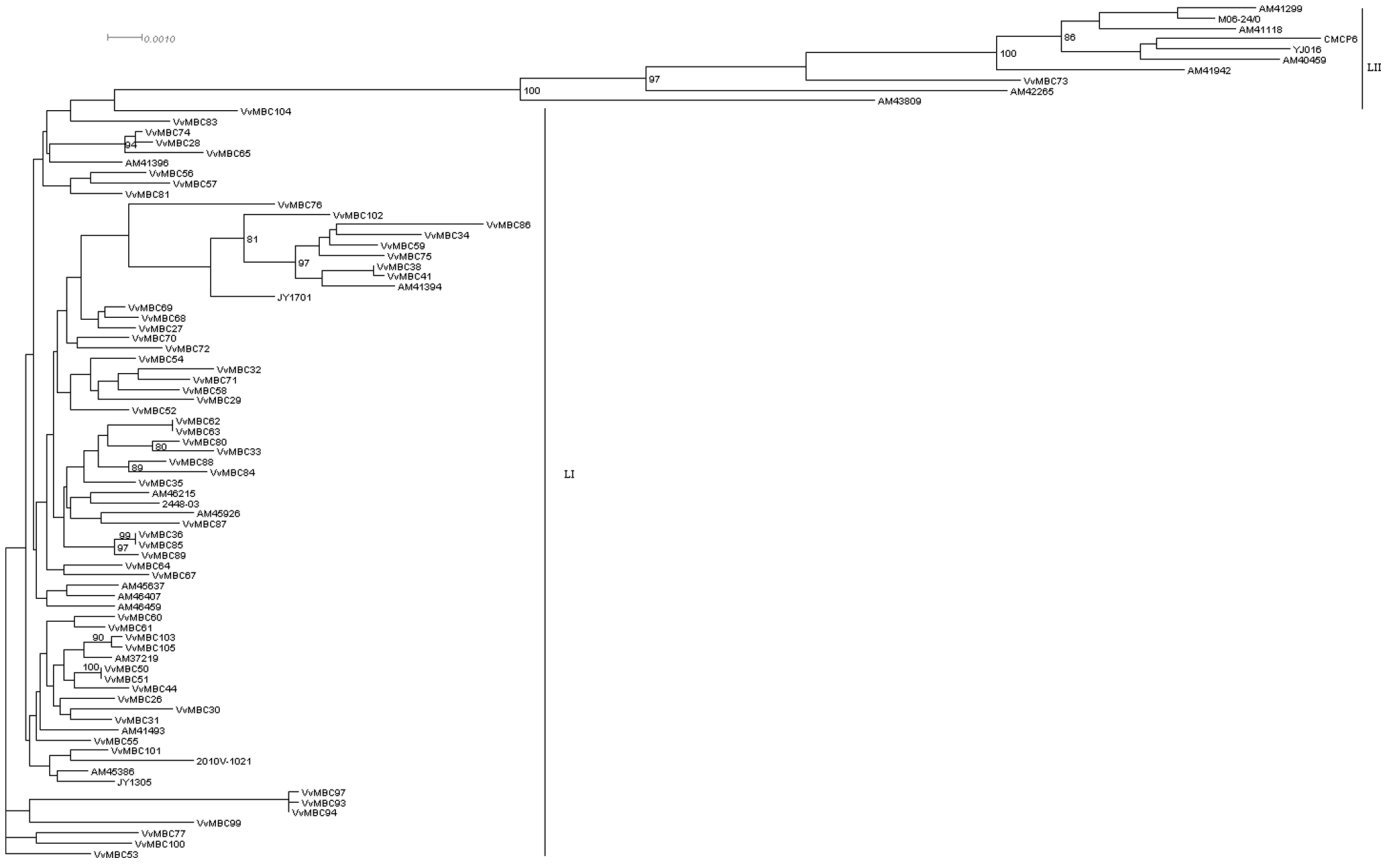
MLST unrooted neighbor joining tree. Dendrogram constructed from the alignment of 3830*rpoD*, *gyrB*, *glp*, *pilF* and *vvhA* for the 83 *V. vulnificus* strains studied using neighbor joining method, Kimura's 2-parameter distance, 1000 bootstraps replicates. Bootstraps above 80 are indicated on the tree.

A total of 3,830 bp, corresponding to a partial sequence of 6 genes (16Sr RNA, *rpoD*, *gyrB*, *glp*, *vvhA* and *pilF*) were aligned, allowing the construction of a concatemer phylogenetic tree by the neighbor joining method and Kimura's 2-parameter distances (Fig. 1). Based upon all 6 genes, 81 profiles were identified among the 83 strains. From the phylogenetic tree, two main lineages can be described: lineages I and II (LI and LII, respectively). Lineage LII contains 10 of the 83 strains studied: the 3 clinical strains completely sequenced (CMCP6, YJ016 and MO6-24O), 6/18 clinical strains provided by the CDC (AM41299, AM41118, AM40459, AM41942, AM42265, AM43809), and only 1/60 environmental strain isolated from oysters in the course of this study in New Jersey (VvMBC73). The LI lineage contains 73 strains: the 2 environmental strains completely sequenced (JY1701 and JY1305), 12/18 clinical strains provided by the CDC, and 59/60 environmental strains isolated from oysters. Consequently LII contains 90% of clinical strains and LI contains 83% of the environmentally-sampled strains. This division into two clusters is typical for *V. vulnificus* and consistent with results obtained from previous studies [11], [14], [16], [41], [42]. Warner and colleagues [17] showed that both genotypes exist in equal proportions in estuarine waters of North Carolina and Florida; whereas, oysters contain mainly (∼84%) *V. vulnificus* strains from LI (called E genotype by Warner and colleagues). In the present study a similar range of values was obtained, with 93% of the strains from oysters belonging to LI. This suggests that LI strains are preferentially ingested by or retained within oysters or that they are better adapted to this niche.

**Figure 2:**
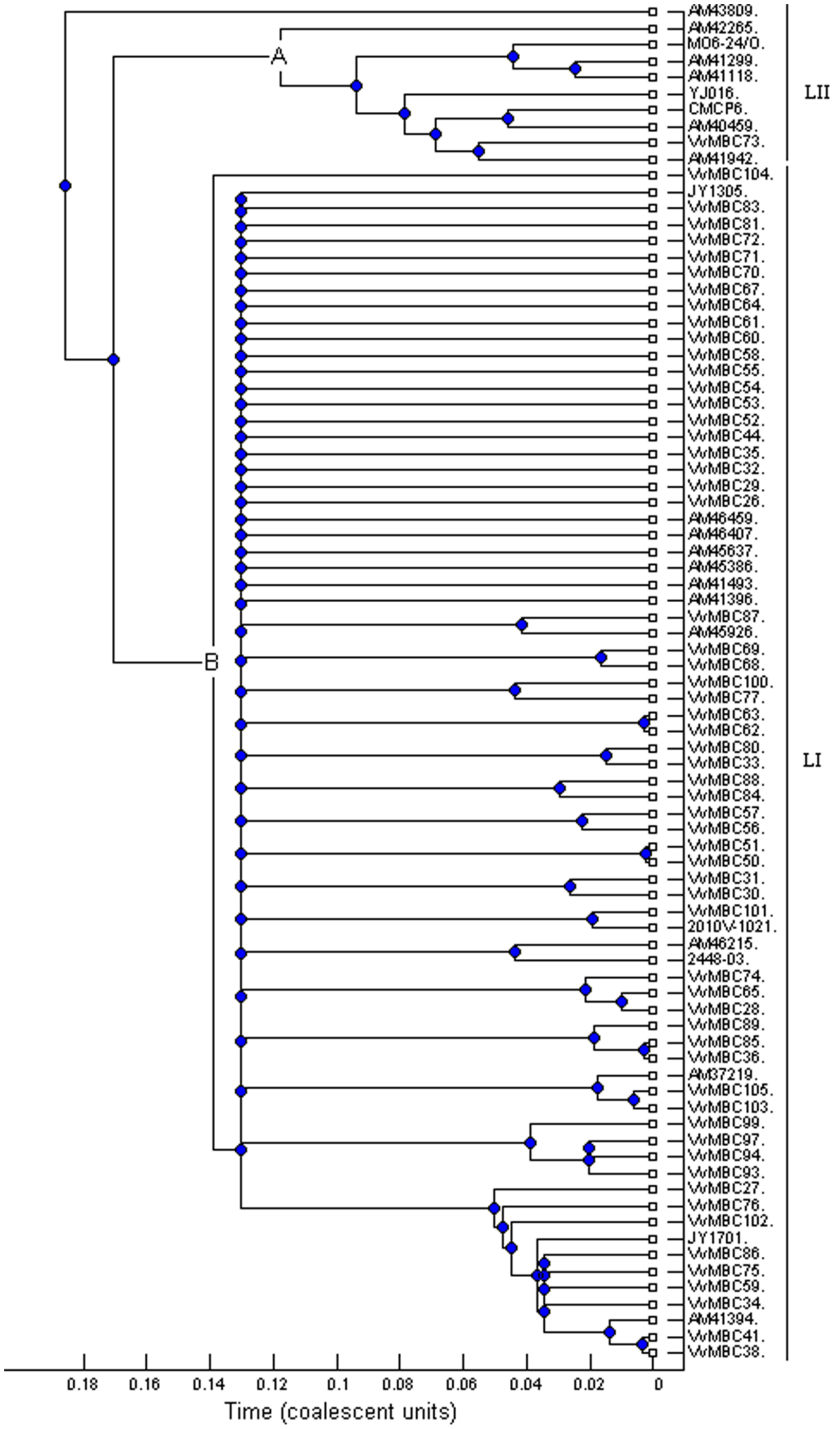
MLST majority-rule consensus tree. Dendrogram based on the genealogies inferred by Clonal Frame after 100000 iterations (including 50000 burn-in iterations), mutation rate θ = 5 and recombination rate ρ = 5.

It has been shown that recombination events greatly complicate the phylogeny of *V. vulnificus* [9], [18], we therefore investigated the relative importance of recombination and mutation in our analyses under clonal frame hypothesis. The 50% majority-rule consensus trees of concatenated sequences were built with initial value of recombination rate ρ = 5 and ρ = 10. Values of θ and ρ were convergent based on the Gelman and Rubin test (datas not shown). The consensus tree (Fig. 2) constructed by Clonal Frame (ρ = 5) highlights a very close topology compared to the null hypothesis of recombination (supporting information Fig. S1) and as compared to the neighbor joining method (Fig. 1) with the two main lineages LI and LII described. Position of the strain AM43809 remains unclear. Clonal Frame estimated that recombination and mutation had approximately the same effect in introducing polymorphism with the ratio r/m = 1.27 (95% credibility interval CI 0.91–1.69) and that recombination happened less frequently than mutation with ρ/θ = 0.42 (95% CI 0.28–0.6). Therefore in our study, recombination seems unlikely to have impacted significantly the phylogeny of *V. vulnificus* isolates compared to point mutation. The inference of ρ/θ and r/m for both lineages shows higher values for LI with r/m = 0.93 (95% CI 0.6–1.44) and ρ/θ = 0.38 (95% CI 0.21–0.63) compared to LII with r/m = 0.15 (95% CI 0.01–0.31) and ρ/θ = 0.014 (95% CI 0.0014–0.03). The evidence of recombination events on the branch anterior to LII (node A) and to LI (node B) is shown graphically on supporting information Fig. S2.

By examining nucleotidic polymorphisms within both lineages (Table 3), LII includes 192 polymorphic sites versus 213 for LI, and nucleotidic diversity π is much higher in LII (0.017) than in LI (0.0084). These results are fully consistent with previous studies [14], [18], with a very similar range of values between lineages. Comparing clinical and environmental strains, it appears that clinical isolates are more diverse with a higher nucleotidic diversity π (0.024) than environmental isolates (0.0093), which has not been shown as clearly before by MLST [14]. These results could indicate a more recent clonal expansion for LI and environmental strains. The ds/dn ratio for clinical strains (28.4) and for environmental strains (41.4) indicates strong purifying selection.

In our study, we did not observe any obvious correlations between the clustering of strains by MLST and geographic origin. However, the MLST sequences provide limited polymorphisms for fine-scale phylogeographic analysis. Our results are consistent with the pattern of sporadic human infection with biotype 1 strains. Globally, a different distribution of commonly studied polymorphic sequences for clinical versus environmental strains was confirmed by PCR screening (Tables 1 and 4). Clinical strains were predominantly 16S rRNA type A (76.2%), CPS type 2 (71.4%), *vcg* type C and E (52.4% and 47.6% respectively). Such a profile appears different than those described before [14], [41] with predominantly 16S rRNA type B, CPS type 1, *vcg* type C. When comparing such results with profiles of clinical strains from LII, we obtained congruent typing: 16S rRNA type B (88.9%), CPS type 1 (55.6%), *vcg* type C (100%). Concerning environmental strains, profiles were predominantly 16S rRNA type A (100%), CPS type 2 (61.7%), *vcg* type E (96.8%) which is congruent with previous studies and which is very similar to clinical strains LI: 16S rRNA type A (100%), CPS type 2 (100%), *vcg* type E (83.3%). We note that only the clinical LII strains have the classical “clinical” profile; whereas, both environmental and clinical LI strains have the classical “environmental” profile. Markers considered here were correlated with lineages described by MLST but not with the clinical or environmental status of the strains.

**Table 4:** Allelic distribution of markers studied in clinical versus environmental *V. vulnificus* strains.

Identifier	Number of isolates (Genotype distribution %)			
	Clinical	Clinical LII	Clinical LI	Environmental
16S type A	16 (76.2%)	1 (11.1%)	12 (100%)	62 (100%)
16S type B	5 (23.8%)	8 (88.9%)	0	0
CPS type 1	5 (23.8%)	5 (55.6%)	0	10 (16.7%)
CPS type 2	15 (71.4%)	3 (33.3%)	12 (100%)	37 (61.7%)
CPS allele absent or not amplified	1 (4.8%)	1 (11.1%)	0	13 (21.7%)
vcg type C	11 (52.4%)	9 (100%)	2 (16.7%)	2 (3.2%)
vcg type E	10 (47.6%)	0	10 (83.3%)	60 (96.8%)
Arylsulfatase A	15 (71.4%)	9 (100%)	6 (50%)	10 (16.1%)
MtlABC	19 (90.5%)	9 (100%)	10 (83.3%)	38 (61.3%)
NanA	20 (95.2%)	9 (100%)	11 (91.6%)	23 (37.1%)

The study of pathogenesis in *V. vulnificus* has not yielded genotypic markers that predict virulence unequivocally (skin infection or septicemia), although some correlations have been observed. Indeed, both lineages LI and LII, and both clinical and environmental strains, have the ability to cause infection in the mouse model [5]. For this reason, a focus should be maintained on relevant, new virulence markers that allow discrimination between pathotypes. Gulig and colleagues [20] identified by SOLiD sequencing of *V. vulnificus* strains representing unique genotype/virulence phenotype combination, 61 genes characteristic of LII strains, i.e. exhibiting a high level of virulence in the iron dextran-treated mouse model. This same study also described an atypical LI strain (99–738 DP-B5) that was also highly virulent. Among those genes putatively linked to virulence, 3 were investigated by PCR in our study: genes coding the arylsulfatase A, MtlABC and NanA. The same genes were identified by Morrison and colleagues as specific to clinically pathogenic strains and absent from environmental strains [19]. Positive arylsulfatase A PCR was obtained in 15 of 21 clinical strains and 10 of 62 environmental strains (Table 1 and 4). Positive *mtlABC* PCR results were obtained for 19 of 21 clinical strains and 38 of 62 environmental strains. Positive *nanA* PCR results were obtained for 20 of 21 clinical strains and 23 of 62 environmental strains; 12 clinical and 11 environmental strains were positive for all 3 genes (Table 1). The first gene coding the arylsulfatase A (sulfate metabolism) is located on the genomic island XII in *V. vulnificus* [14] and has been described as being associated with virulence of clinical strains. Activity of the enzyme Arylsulfatase A could help to provide a pathogen with sulfur within the host, thereby enhancing survival in sulfur-limited environments. The second gene, *mtlABC*, encoding the mannitol/fructose-specific phosphotransferase system IIA protein, appears to be linked with virulence, although its precise role in virulence remains unknown; the ability to ferment mannitol appears to be more common in the LII lineage [43]. Finally, the gene *nanA* coding the N-acetylneuraminate lyase involved in sialic acid catabolism, was also amplified; the ability to metabolize sialic acid was shown to be essential for *V. vulnificus* virulence [44]; a correlation between the presence of sialic acid catabolism cluster (SAC) and LII (and then clinical strains) was also highlighted before [45]. Our results confirm this relationship in the strains we studied with same range of values.

Phylogenetic trees based upon sequence analysis of these three virulence markers led to essentially the same kind of pattern of clustering obtained by MLST. The phylogenetic tree based upon sequences of arylsulfatase A and obtained by neighbor joining method in one hand and by accounting for recombinations using Clonal Frame in the other hand (supporting information Fig. S3 and S4 respectively) resulted in very close clustering as MLST results except for strain AM42265. Here recombination does not seem to have impacted significantly the phylogeny of *V. vulnificus* isolates compared to point mutation. For MtlABC trees (supporting information Fig. S5 and S6), clustering are close each other with all strains belonging to LII according to MLST clustering together with 5 more clinical strains. Concerning the NanA trees (supporting information Fig. S7 and S8) the isolates seem to be related in a complicated genealogy different than lineages obtained by MLST and that can not be readily resolved here. We can conclude that on the Clonal Frame tree, a first cluster contains a majority of environmental isolates (16/19) and the second cluster a majority of clinical isolates (17/24). Further experiments should be performed on a wider collection of *V. vulnificus* strains to confirm the relevance of those three genes for utilization in phylogenetic analyses.

Three findings of this and other studies suggest that some environmental isolates from our study may have the ability to infect humans: 1) virulence markers were identified in clinical and environmental strains, 2) both clinical and environmental strains are represented in both lineages, and 3) environmental strains can be virulent in experimental infection on the subcutaneously-inoculated iron dextran-treated mouse model [5], [19]. We hypothesize that a strain such as VvMBC73, which clustered in MLST with LII clinical strains (already tested for high virulence using the mouse bioassay), characterized by a *vcg* type C, CPS type 2 and positive amplifications by PCR of arylsulfatase A, *mtlABC* and *nanA* genes, has an overall profile of a strain capable of causing a *V. vulnificus* infection in a human. Nevertheless, virulence assays are required to determine the virulence potential of environmental strains phylogenetically close to clinical ones. To the best of our knowledge, this is the first polyphasic molecular typing study of *V. vulnificus* strains in the northeastern USA.

In the near future, with the development of high throughput genome sequencing, comparison of the sequences of *V. vulnificus* bacterial collections with all combinations of genotypes/pathotypes should lead to the identification of genes, or SNP (Single nucleotide polymorphism), markers that are diagnostic of highly-virulent strains capable of human infection. Finally such approaches with high resolving power should be applied to an epidemiological survey of *V. vulnificus*. In the meantime, it is reasonable to take precautions seasonally in post-harvest treatment of oysters and other shellfish (e.g., shipboard icing) harvested from the entire east coast of the USA based upon the expectation that pathogenic strains may be present anywhere on this coast.

## Supporting Information

Figure S1 **MLST majority-rule consensus tree based on the genealogies inferred by Clonal Frame with the null hypothesis of recombination (ρ = 0).**
(TIF)

Figure S2 **Genetic representation of events indicated in** **Fig. 2** **nodes A and B.** Columns correspond to six MLST gene fragments. Black crosses indicate inferred substitutions with the intensity proportional to its probability and the height of the red lines represents the inferred probability for recombination on a scale from 0 to 1 (*Y*-axis).
(TIF)

Figure S3 **Arylsulfatase A unrooted neighbor joining tree; Kimura's 2-parameter distance, 1000 bootstraps replicates.**
(TIF)

Figure S4 **Arylsulfatase A majority-rule consensus tree based on the genealogies inferred by Clonal Frame after 100000 iterations (including 50000 burn-in iterations), mutation rate θ = 5 and recombination rate ρ = 5.**
(TIF)

Figure S5 **MtlABC unrooted neighbor joining tree; Kimura's 2-parameter distance, 1000 bootstraps replicates.**
(TIF)

Figure S6 **MtlABC majority-rule consensus tree based on the genealogies inferred by Clonal Frame after 100000 iterations (including 50000 burn-in iterations), mutation rate θ = 5 and recombination rate ρ = 5.**
(TIF)

Figure S7 **NanA unrooted neighbor joining tree; Kimura's 2-parameter distance, 1000 bootstraps replicates.**
(TIF)

Figure S8 **NanA majority-rule consensus tree based on the genealogies inferred by Clonal Frame after 100000 iterations (including 50000 burn-in iterations), mutation rate θ = 5 and recombination rate ρ = 5.**
(TIF)
