# Supplementary figures and images for: Molecular Typing of Environmental and Clinical Strains of Vibrio vulnificus Isolated in the Northeastern USA
Source: PLoS One. 2013 Dec 30;8(12):e83357. doi: 10.1371/journal.pone.0083357 (PMC3875459; doi:10.1371/journal.pone.0083357)

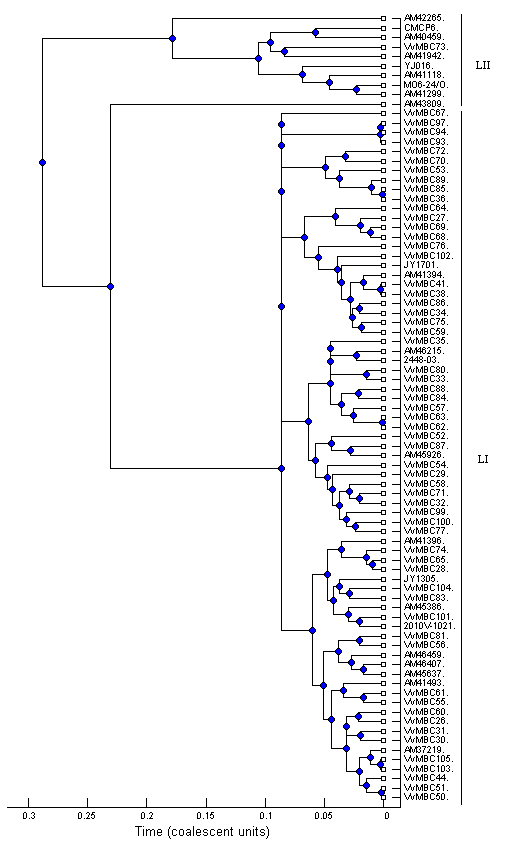

Supplement: Figure S1 — MLST majority-rule consensus tree based on the genealogies inferred by Clonal Frame with the null hypothesis of recombination (ρ = 0). (TIF) [file pone.0083357.s001.tif]

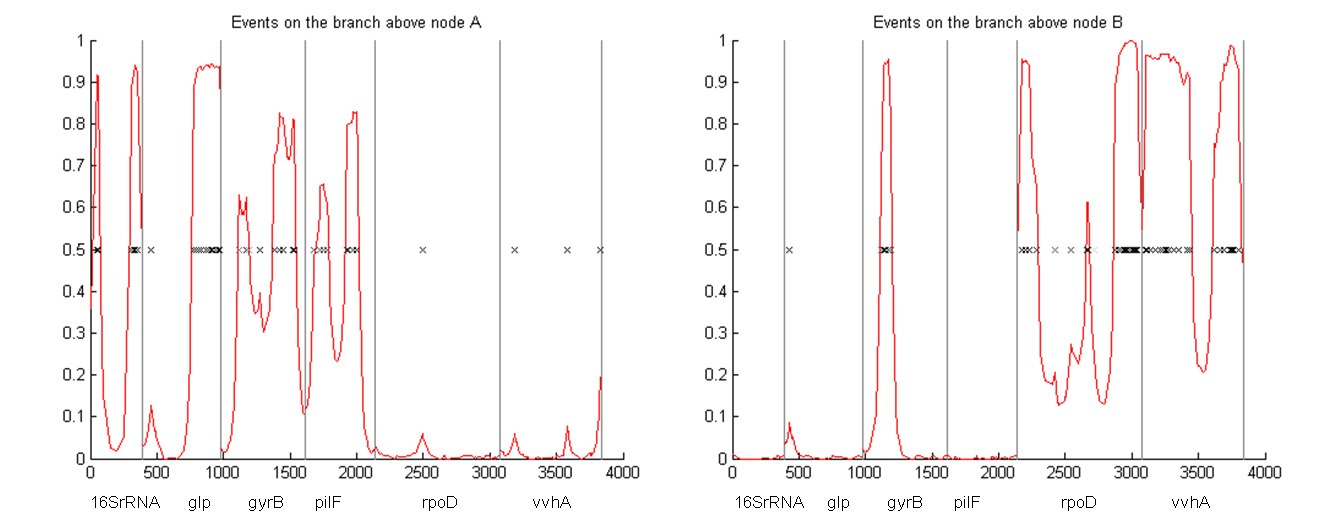

Supplement: Figure S2 — Genetic representation of events indicated in Fig. 2 nodes A and B. Columns correspond to six MLST gene fragments. Black crosses indicate inferred substitutions with the intensity proportional to its probability and the height of the red lines represents the inferred probability for recombination on a scale from 0 to 1 (Y-axis). (TIF) [file pone.0083357.s002.tif]

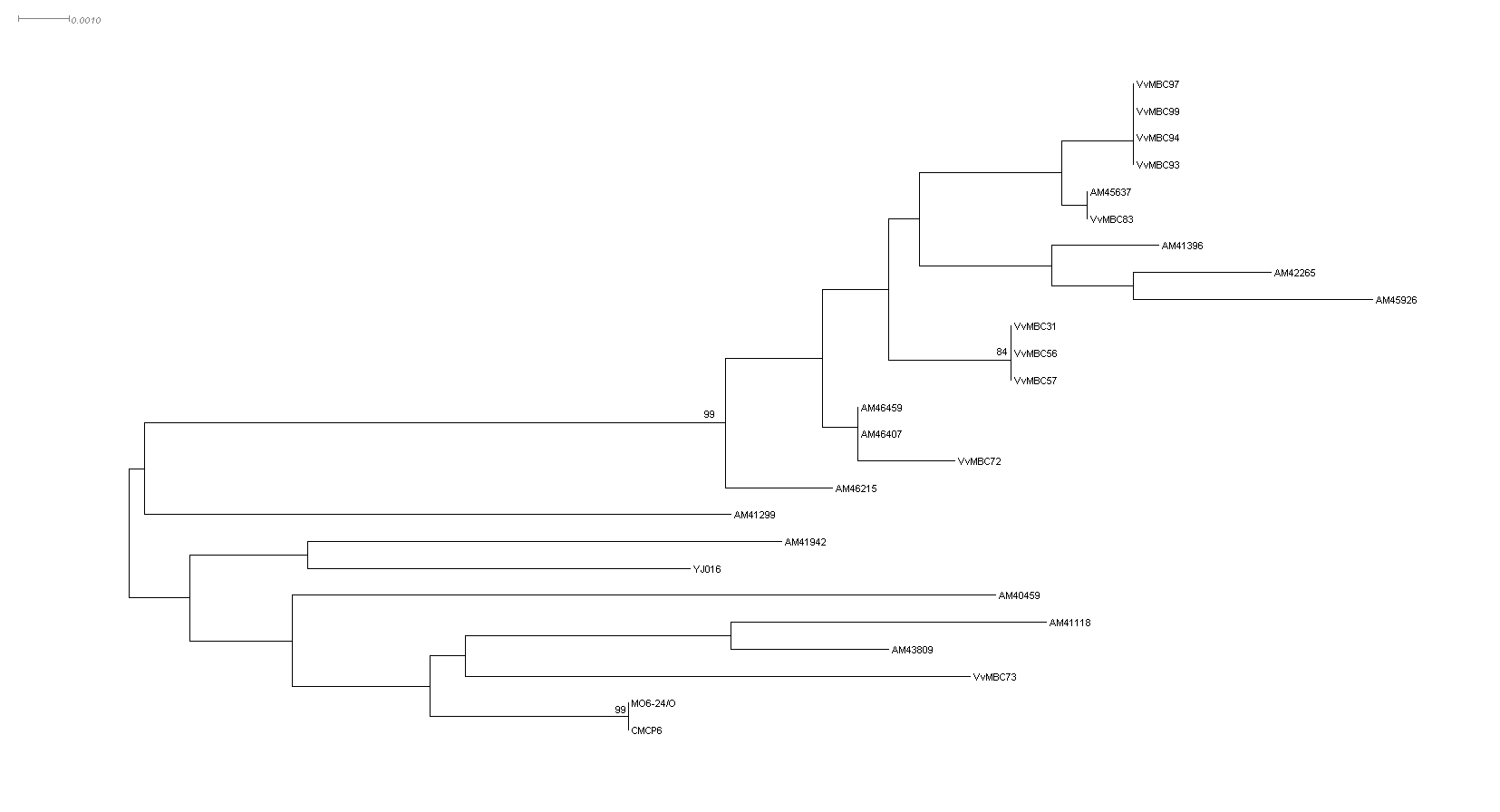

Supplement: Figure S3 — Arylsulfatase A unrooted neighbor joining tree; Kimura's 2-parameter distance, 1000 bootstraps replicates. (TIF) [file pone.0083357.s003.tif]

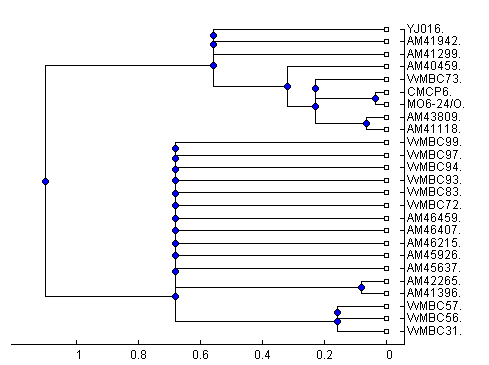

Supplement: Figure S4 — Arylsulfatase A majority-rule consensus tree based on the genealogies inferred by Clonal Frame after 100000 iterations (including 50000 burn-in iterations), mutation rate θ = 5 and recombination rate ρ = 5. (TIF) [file pone.0083357.s004.tif]

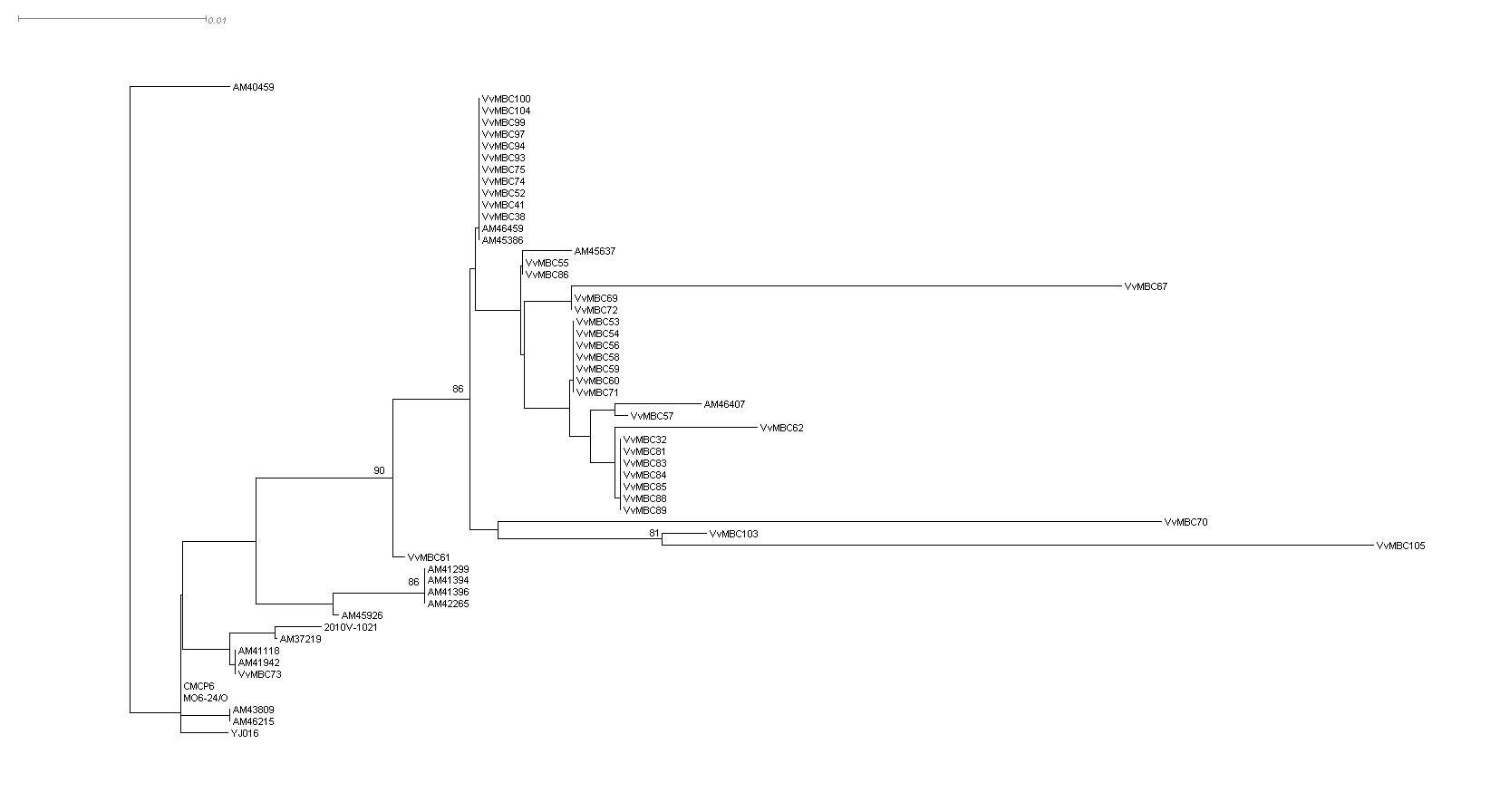

Supplement: Figure S5 — MtlABC unrooted neighbor joining tree; Kimura's 2-parameter distance, 1000 bootstraps replicates. (TIF) [file pone.0083357.s005.tif]

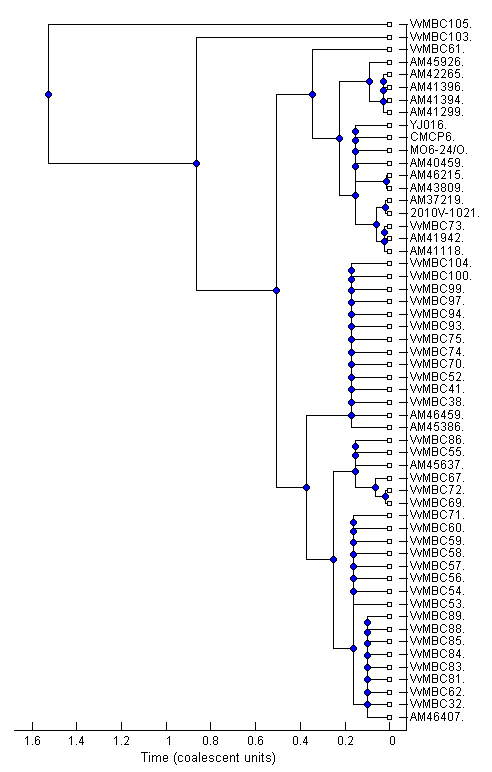

Supplement: Figure S6 — MtlABC majority-rule consensus tree based on the genealogies inferred by Clonal Frame after 100000 iterations (including 50000 burn-in iterations), mutation rate θ = 5 and recombination rate ρ = 5. (TIF) [file pone.0083357.s006.tif]

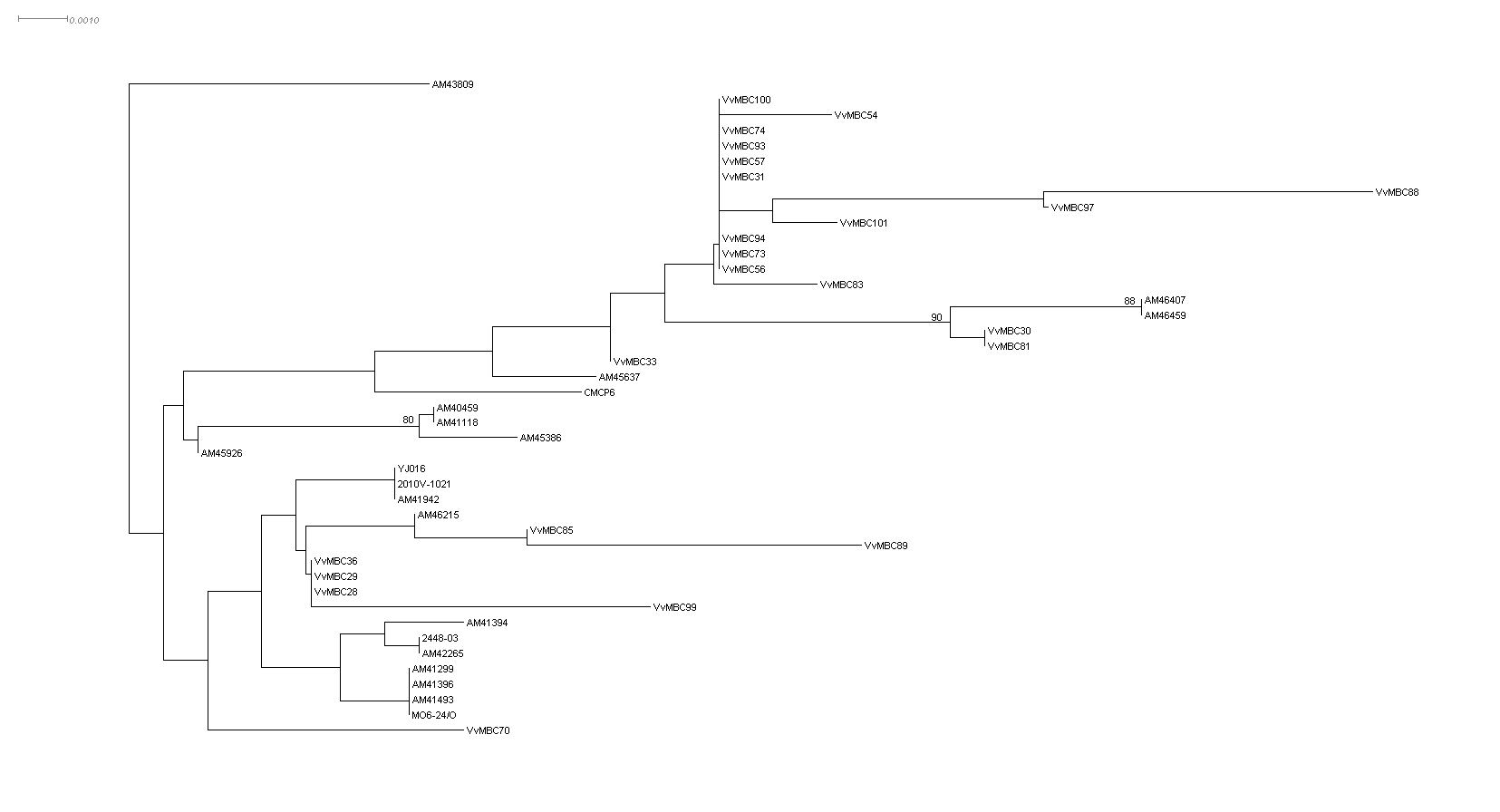

Supplement: Figure S7 — NanA unrooted neighbor joining tree; Kimura's 2-parameter distance, 1000 bootstraps replicates. (TIF) [file pone.0083357.s007.tif]

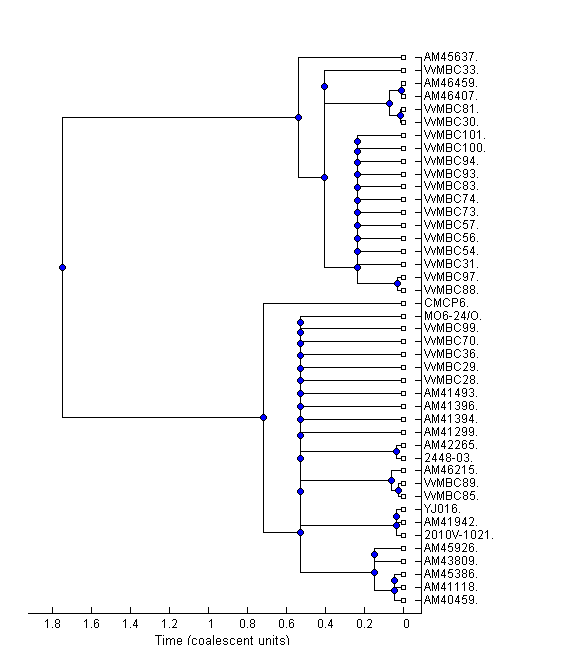

Supplement: Figure S8 — NanA majority-rule consensus tree based on the genealogies inferred by Clonal Frame after 100000 iterations (including 50000 burn-in iterations), mutation rate θ = 5 and recombination rate ρ = 5. (TIF) [file pone.0083357.s008.tif]
